# Lipid Membrane Adsorption Determines Photodynamic Efficiency of β-Imidazolyl-Substituted Porphyrins

**DOI:** 10.3390/biom9120853

**Published:** 2019-12-10

**Authors:** Irene Jiménez-Munguía, Arseniy K. Fedorov, Inna A. Abdulaeva, Kirill P. Birin, Yury A. Ermakov, Oleg V. Batishchev, Yulia G. Gorbunova, Valerij S. Sokolov

**Affiliations:** 1 National University of Science and Technology “MISiS”, 4 Leninskiy pr. 119049 Moscow, Russia; 2 A. N. Frumkin Institute of Physical Chemistry and Electrochemistry, Russian Academy of Sciences, 31/4 Leninskiy pr. 119071 Moscow, Russia; fedorov@gmail.com (A.K.F.); Abdulaeva@gmail.com (I.A.A.); kirill.birin@gmail.com (K.P.B.); yury.a.ermakov@gmail.com (Y.A.E.); olegbati@gmail.com (O.V.B.); sokolov.valerij@gmail.com (V.S.S.); 3 Moscow Institute of Physics and Technology, 9 Institutskiy Lane, Dolgoprudniy, 141700 Moscow Region, Russia; 4 N. S. Kurnakov Institute of General and Inorganic Chemistry, Russian Academy of Sciences, 31 Leninskiy pr. 119119 Moscow, Russia

**Keywords:** photosensitizers, porphyrins, lipid membrane, adsorption, singlet oxygen, styryl dyes

## Abstract

Photosensitizers (PSs) represent a group of molecules capable of generating reactive oxygen species (ROS), such as singlet oxygen (SO); thus, they are considered to be promising agents for anti-cancer therapy. The enhancement of the photodynamic efficiency of these compounds requires increasing the PS activity in the cancer cell milieu and exactly at the target cells. In the present work, we report the synthesis, lipid membrane binding and photodynamic activity of three novel cationic PSs based on β-imidazolyl-substituted porphyrin and its Zn(II) and In(III) complexes (1H2, 1Zn and 1In). Comparison of the behavior of the investigated porphyrins at the bilayer lipid membrane (BLM) demonstrated the highest adsorption for the 1In complex and the lowest one for 1Zn. The photodynamic efficiency of these porphyrins was evaluated by determining the oxidation rate of the styryl dye, di-4-ANEPPS, incorporated into the lipid membrane. These rates were proportional to the surface density (SD) of the porphyrin molecules at the BLM and were roughly the same for all three porphyrins. This indicates that the adsorption of these porphyrins at the BLM determines their photodynamic efficiency rather than the extinction or quantum yield of singlet oxygen.

## 1. Introduction

Photodynamic therapy (PDT) is a widely used method for the treatment of skin cancer [1,2,3,4,5] and also suggested as a tool for the killing of drug-resistant bacteria [6,7,8,9,10]. The key factors in PDT are photosensitizers (PSs) capable of generating reactive oxygen species (ROS) upon excitation with visible light. Singlet oxygen (SO) is the most prominent and stable example of ROS. SO molecules initiate cell death by the oxidation of proteins, lipids, nucleic acids and carbohydrates of the target cell [2]. Development of PDT techniques requires the synthesis and testing of novel PSs with more efficient and targeted action [1,2,5]. It should be noted that the great impact on the efficiency of PS is related to their ability to precisely bind to the plasma membrane of cancer cells. 

One of the best model systems allowing in vitro investigation of the PS efficiency is a bilayer lipid membrane (BLM), which models the lipid matrix of cellular membranes. A BLM offers the possibility of study the PS binding and evaluate its photodynamic efficiency by measuring the oxidation rate of the target molecules incorporated into the membrane with SO [11,12,13,14,15,16]. 

Recently, we have developed a new approach for the investigation of the efficiency of PSs on BLM based on measurements of boundary potentials at the membrane/water interface [17,18]. Using this approach, we have studied sulfonate-substituted aluminum phthalocyanine and 5,10,15,20-tetrakis(p-sulfonatophenyl)porphyrin as photosensitizers [18,19,20] and have revealed several problems decreasing the efficiency of PSs, namely, the quenching of SO by phthalocyanines in the membrane [13,20] and weak binding of 5,10,15,20-tetrakis(*p*-sulfonatophenyl)porphyrin (TPPS4) to the BLM at low pH due to aggregation [19]. In this regard, porphyrins with positively charged peripheral groups seem to be the most promising molecules for PDT [21,22,23,24]. Due to positive charge, such porphyrins pass through the cellular membranes more readily and bind to DNA molecules by intercalation [25].

**Figure 1:**
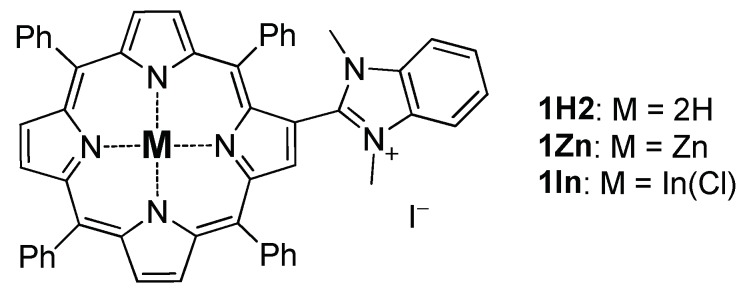
Chemical structures of 1H2, 1Zn and 1In porphyrins.

Herein, we report the synthesis, membrane binding and photodynamic activity of three novel cationic PSs based on β-imidazolyl-substituted porphyrin and its Zn(II) and In(III) complexes (1H2, 1Zn and 1In) (Figure 1). This investigation will contribute to a better understanding of the molecular mechanisms occurring at the membrane surface during photodynamic therapy. 

## 2. Materials and Methods 

### 2.1. Synthesis of the Porphyrins

All chemicals were purchased from commercial suppliers, unless otherwise stated. The solvents were purified according to conventional methods [26]. Chromatographic purifications were performed with Macherey-Nagel, Silica 60, 0.063–0.2 mm. Merck aluminum plates (TLC Silica 60 F254, Darmstadt, Germany) were used for the TLC analysis, which was performed with dichloromethane/hexane mixtures as eluents.

^1^H-NMR spectra of 10^–4^ M solutions in CDCl_3_ were recorded on a Bruker Avance III spectrometer (Billerica, MA, USA) at a frequency of 600 MHz at 303 K, using the resonance of the residual CHCl_3_ as an internal reference (δ = 7.26 ppm). MALDI-TOF mass spectra were recorded on a Bruker Daltonics Ultraflex spectrometer (Billerica, MA, USA) in the positive ion mode without a matrix. UV–vis spectra were recorded on a Thermo Scientific Helios Alpha spectrophotometer (Waltham, MA, USA) in the 250 to 900 nm range using rectangular quartz cells with an optical path length of 0.0882–10 mm. 

The free-base 5,10,15,20-tetraphenyl-2-(benzimidazol-2-yl)-porphyrin and 5,10,15,20-tetraphenyl-2-(N,N-dimethylbenzimidazol-2-yl)-porphyrin were prepared following the published procedure [27]. 

#### 2.1.1. Zinc(II) 5,10,15,20-Tetraphenyl-2-(N, N-Dimethylbenzimidazol-2-Yl)-Porphyrin

The Zn(II) complex was prepared by the interaction of the free-base 5,10,15,20-tetraphenyl-2-(2-benzimidazolyl)-porphyrin with 5 equiv. of Zn(OAc)_2_ following the typical procedure [28]. 1H2 (25 mg, 0.03 mmol) was dissolved in CHCl_3_/MeOH mixture (4/1, 50 mL) and Et_2_NH (50 μL) was added. Solid Zn(OAc)_2_ (27 mg, 0.15 mmol, 5 equiv.) was added and the mixture was kept at ambient temperature upon stirring until complete conversion of the starting material (ca. 3 h). Afterwards water (50 mL) was added, and the mixture was transferred to a separation funnel and extracted. The organic layer was separated and evaporated to dryness to provide pure 1Zn. Yield 26 mg, 98%.

MALDI-TOF MS: calculated for C_53_H_37_N_6_Zn [M]^+^ 821.2, found 821.5.

UV-Vis (CHCl_3_; λ_max_ (nm); log(ε)): 432 (5.42), 564 (4.13), 611 (4.07).

^1^H-NMR (CDCl_3_/MeOD = 1/1 + 2 equiv. DABCO; δ, ppm; ^n^*J*, Hz): 9.14 (s, 1H, H_β_), 8.86 (d, 1H, ^3^*J* = 4.7, H_β_), 8.84–8.80 (m, 3H, H_β_), 8.73 (d, 1H, ^3^*J* = 4.7, H_β_), 8.52 (d, 1H, *J* = 4.7, H_β_), 8.25 (d, 2H, ^3^*J* = 7.2, H_o-Ph_), 8.18 (d, 2H, ^3^*J* = 7.2, H_o-Ph_), 8.14 (d, 2H, ^3^*J* = 7.2, H_o-Ph_), 8.03 (d, 2H, ^3^*J* = 7.2, H_o-Ph_), 7.77–7.67 (m, 9H, H_m_-_Ph_+H_p-Ph_), 7.67–7.64 (m, 2H, H_Bz_), 7.64–7.61 (m, 2H, H_Bz_), 7.15 (t, 2H, ^3^*J* = 7.6, H_m-Ph_), 6.83 (t, 1H, ^3^*J* = 7.7 Hz, H_p-Ph_), 3.82 (s, 6H, Me) (Figure S1).

#### 2.1.2. Chlorooindium(III) 5,10,15,20-Tetraphenyl-2-(N, N-Dimethylbenzimidazolium-2-Yl)-Porphyrin Iodide

The synthesis was based on the published protocol [29]. The free-base 5,10,15,20-tetraphenyl-2-(benzimidazol-2-yl)-porphyrin (73 mg, 0.1 mmol), InCl_3_ (110 mg, 0.5 mmol) and NaOAc (410 mg, 5 mmol) were refluxed in AcOH (5 mL) for 1.5 h. The conversion was monitored by TLC, and the reaction was continued until the starting porphyrin was completely consumed. After cooling to ambient temperature, the reaction mixture was diluted with CHCl_3_ (50 mL) and extracted with water (50 mL). The organic phase was separated, evaporated to dryness and applied to a column packed with silica gel in CHCl_3_. The column was eluted with CHCl_3_/MeOH mixtures (0→6% of MeOH) containing 0.2% of Et_2_NH. The fraction of the target complex was eluted with 3→6% of MeOH in CHCl_3_ and evaporated to provide 96 mg (96%) of the chloroindium(III) complex, which was subsequently used in the next synthetic step. 

The chloroindium(III) 5,10,15,20-tetraphenyl-2-(benzimidazol-2-yl)-porphyrin (96 mg, 0.1 mmol) was mixed with MeI (200 μL, 3.2 mmol) and K_2_CO_3_ (200 mg, 1.5 mmol) in dry DMF (5 mL) and heated at 100 °C under argon overnight. Afterwards, the reaction mixture was cooled to ambient temperature, diluted with ethyl acetate (50 mL) and extracted with water (3 × 50 mL). The organic layer was evaporated to dryness, applied to a column packed with silica gel in DCM and eluted with DCM/MeOH mixtures (0→5% of MeOH). The collected green fraction of the target chloroindium(III) 5,10,15,20-tetraphenyl-2-(N,N-dimethylbenzimidazolium-2-yl)-porphyrin iodide was evaporated and repeatedly purified on a Bio-Beads SX-1 column with a CHCl_3_/EtOH (2.5% of EtOH) eluent. The evaporation of the obtained fraction provided 33 mg (32%) of the target complex.

^1^H-NMR (CDCl_3_; δ, ppm; ^n^*J*, Hz): 9.75 (s, 1H, H_β_), 9.13 (d, 1H, ^3^*J* = 4.7, H_β_), 9.06 (d, 1H, ^3^*J* = 4.7, H_β_), 9.00 (d, 1H, ^3^*J* = 4.7, H_β_), 8.77 (d, 1H, *J* = 4.7, H_β_), 8.30–8.34 (br.m.,1H, H_o-Ph_), 8.25 (d, 2H, ^3^*J* = 7.2, H_o-Ph_), 8.22 (d, 2H, ^3^*J* = 6.8, H_o-Ph_), 8.05 (d, 2H, ^3^*J* = 7.3, H_o-Ph_), 7.76–7.89 (m, 12H, 10H_Ph_ + 2H_Bz_), 7.72 (dd, 2H, ^3^*J* = 6.0, ^4^J = 3.4, H_Bz_), 7.32 (t, 2H, ^3^*J* = 7.6, H_m-Ph_), 6.97 (t, 1H, ^3^*J* = 7.7 Hz, H_p-Ph_), 3.95 (s, 6H, Me) (Figures S1–S4).

MALDI-TOF MS: calculated for C_53_H_37_InN_6_ [M-Halogen]^+^ 872.2, found 872.5.

HR-MS: calculated for C_53_H_37_ClInN_6_ [M]^+^ 907.1802, found 907.1888 (Figure S5).

UV-Vis (CHCl_3_; λ_max_ (nm); log(ε)): 316 (4.31), 435 (5.57), 569 (4.18), 613 (4.14).

### 2.2. Experiments at BLM

BLMs were formed by the Mueller-Rudin technique [30] from a 15 mg/mL solution of diphytanoyl phosphatidylcholine (Avanti polar lipids, Alabaster, AL, USA) in n-decane (Sigma-Aldrich, Saint-Louis, MO, USA) at an aperture diameter of about 0.8 mm in a Teflon septum that separated two aqueous compartments of equal volumes (2 mL) stirred by a magnetic stirrer. Membrane bathing solutions were prepared with KCl, citric acid (“Reachim”, Moscow, Russia), TRIS and HEPES (Sigma-Aldrich, Saint-Louis, MO, USA) dissolved in double-distilled water. Porphyrins and the styryl dye di-4-ANEPPS (Sigma-Aldrich, Saint-Louis, MO, USA) were added into one compartment from stock solutions in ethanol. The total concentration of ethanol in water never exceeded 3%. The membrane was illuminated by a semiconductor laser (wavelength 405 nm, optic power 1 mW), and porphyrins were introduced into the compartment distant from the light source. The measurements of absorption spectra of the porphyrins in ethanol and in water solutions at various pH were performed on a Panorama Fluorat 02 (Lumex, Saint Petersburg, Russia) fluorescence spectrophotometer. 

The setup for electrical measurements was similar to that described by us earlier [18,19]. Measurements were performed with the aid of a pair of Ag/AgCl electrodes with agar bridges. The bridges were made of standard plastic pipette tips, the bottom part of which was filled with agarose gel, and the remaining volume was filled with a 0.1 M KCl solution. Total electrical resistance of the electrodes with the bridges did not exceed 50 kΩ. The membrane capacitance and conductance were continuously measured by a technique similar to that described in [17,18]. The change of the boundary potential difference (Δφ_b_) due to adsorption of porphyrins on the BLM was measured by two methods whose combination makes it possible to study both the adsorption of substances on the membrane and their penetration through the BLM. The first one, the inner field compensation (IFC) method, is based on the measurement of the second harmonics of the capacitive current ([31]). In the second method, the change of Δ*φ_b_* is determined from the change of the BLM conductance induced by ionophore nonactin as [32,33,34,35]

(1) $$\Delta \varphi_{b}=-\frac{RT}{zF}\ln\left(\frac{g}{g_{0}}\right),$$

where *g*_0_ and g are the conductance before and after the addition of porphyrins, respectively; z = +1 is the charge number of the nonactin-K^+^ complex; and F, R and T are the Faraday number, gas constant and absolute temperature, respectively.

To estimate the surface concentration of porphyrin molecules at the membrane, we determined the change of the membrane surface charge caused by their adsorption, using the Gouy–Chapman equation relating the membrane surface potential to its surface charge density [19,20]. The change in membrane surface potential (ζ-potential) upon adsorption of porphyrins was measured via electrophoretic mobility of liposomes by dynamic light scattering using a Zetasizer II (Malvern Instruments, Malvern, UK) supplied with a correlator (PhotoCor SP, College Park, MD, USA). Liposomes were prepared by vacuum drying of a solution of diphytanoyl phosphatidylcholine (Avanti polar lipids, Alabaster, AL, USA) in chloroform in a round bottom glass flask in a rotary evaporator for about 50 min with subsequent resuspension in a water buffer solution and shaking of the resulting sample (BioVortex V-1, Biosan, Riga, Latvia). The final concentration of lipids in the solution was 1 mg/mL. The spectrum of electrophoretic mobility was calculated using the software developed by the authors on the basis of the Malvern algorithm. The value of the ζ-potential was calculated from the mobility using the Smoluchowski equation. 

## 3. Results and Discussion

### 3.1. Adsorption on the Membrane

**Figure 2:**
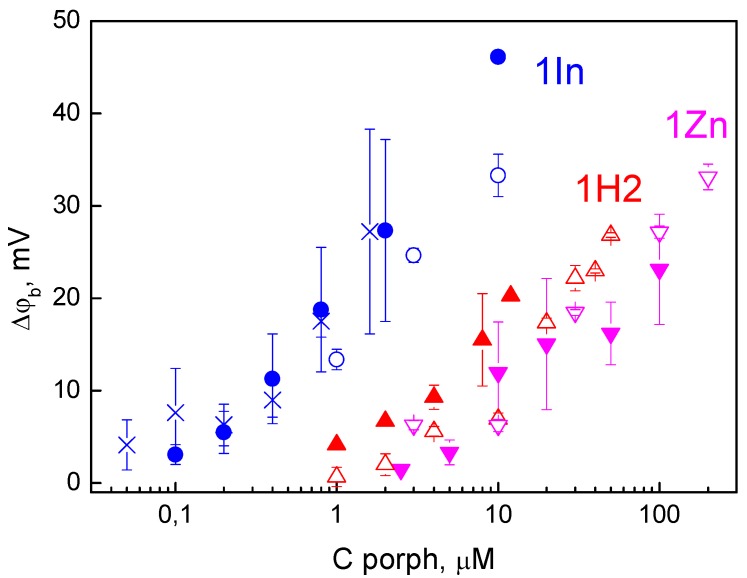
Adsorption of porphyrins on the lipid membrane. The boundary potential changes due to the adsorption of three porphyrins were determined either by the inner field compensation (IFC) method (solid symbols), or as the ζ-potential change of liposomes (blank symbols), or from the nonactin-induced change of conductance (crosses). Measurements were performed in 20 mM KCl, 2 mM HEPES, pH 7.0 buffer solutions.

Adding the ethanol solution of porphyrins to a bathing water solution at one side of the BLM resulted in a change of the boundary potential difference, Δφ_b_, indicating their adsorption on the membrane. The dependence of Δφ_b_ on the concentration of porphyrins in a water solution is presented in Figure 2. The comparison of three porphyrins showed that the highest Δφ_b_ values were revealed in the case of 1In, while the lowest values were achieved for the 1Zn porphyrin. The sign of the Δφ_b_ corresponds to the adsorption of positively charged molecules, which is in agreement with the structures of the porphyrins containing positively charged benzimidazole peripheral groups. Dependences of ζ-potentials on the concentration of the porphyrins are shown in Figure 2. The values of ζ-potentials were equal or slightly lower than the Δφ_b_ values, indicating the absence or shallow insertion of the charged groups of the porphyrin molecules into the lipid membrane [34,35]. The same behavior was observed earlier for the negatively charged porphyrins [19]. The difference in Δφ_b_ for three porphyrins can be explained by affinity of these compounds to the lipid bilayer. As it was shown by us earlier using phthalocyanines, this affinity depends on the nature of the metal cation inside the molecule, because the adsorption of such compounds at BLM is determined by the coordination bond between the metal cation and phosphate group of phospholipids [15]. Perhaps, the same mechanism is valid for the porphyrins.

### 3.2. Photodynamic Efficiency 

The photodynamic efficiency of the porphyrins was studied similar to the reported procedure [18]. In brief, we measured the oxidation rate of the styryl dye di-4-ANEPPS, which served as a target molecule (TM) for SO. di-4-ANEPPS was added into the water solution either at the same side of the membrane with the porphyrin (*cis*) or at the opposite side (*trans*). The adsorption of the di-4-ANEPPS molecules on the BLM led to an increase in the dipole potential on its surface. The illumination of the BLM with adsorbed di-4-ANEPPS and porphyrin molecules leads to a decrease of this potential due to the oxidation of di-4-ANEPPS molecules. The oxidation rate, *R*, is the measure of the steady-state concentration of SO generated by the porphyrin molecules under illumination [18]. The value of *R* was calculated from the kinetics of the decrease of the potential during illumination and its restoration after switching the light off [18]:

(2) $$R={\frac{d\varphi_{rel}\left(t\right)}{dt}|}_{t=0},\;\mathrm{or}\;R=\frac{1}{\tau_{L}}-\frac{1}{\tau_{D}},$$

where $\varphi_{rel}\left(t\right)=\frac{\varphi \left(t\right)}{\varphi_{ads}}$. *φ_ads_* is the boundary potential difference arising due to adsorption of di-4-ANEPPS prior to the illumination; *φ*(*t*) is the potential difference measured after the beginning of the illumination; and *τ_L_* and *τ_D_* are time constants of exponents approximating the kinetics of *φ*(*t*) during the illumination and the following dark stages, respectively. 

**Figure 3:**
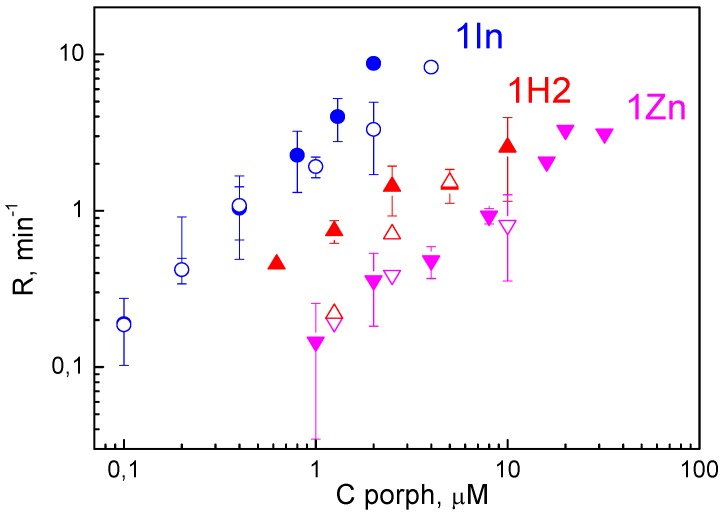
Dependence of the oxidation rate (*R*) of di-4-ANEPPS on the concentration of porphyrins in the solution. The target molecules of di-4-ANEPPS were adsorbed either on the *cis* side (solid symbols) or on the *trans* side (blank symbols) of the membrane. The solution contained 20 mM KCl, 2 mM HEPES, pH 7. Di-4-ANEPPS was added in a concentration of 0.3 μM.

The dependences of *R* on the concentrations of porphyrins in the solution are presented in Figure 3. The values of *R* for the *cis* arrangement of the TM was either the same or insignificantly higher than that for the *trans* arrangement. The comparison of the *R* values for three porphyrins shows that their photodynamic efficiency changes along with the boundary potential difference induced by their adsorption: the higher *R* values were for 1In, and the lower ones were for 1Zn. This indicates that the difference in the efficiency for three porphyrins could correlate with their affinity to BLM. To prove this hypothesis we calculated the surface charge density for adsorbed porphyrin molecules using ζ-potential data and the Gouy–Chapman equation [19]. 

**Figure 4:**
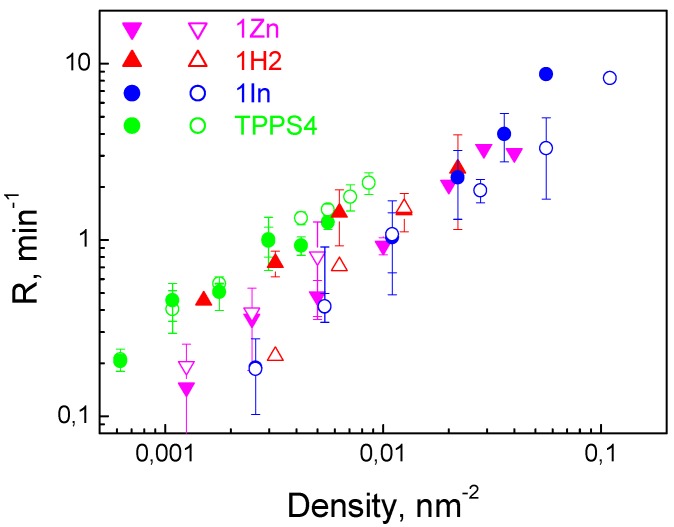
Dependence of the oxidation rate of di-4-ANEPPS on the surface density of porphyrin molecules at the membrane. The data points were taken from Figure 3. Surface density was calculated from the change in the ζ-potential using the Gouy–Chapman equation. Green symbols are the data obtained early for TPPS4 [19].

Taking the charge of the porphyrin equal to the charge of one proton provided by the peripheral benzimidazolium group at neutral pH, we could go from the surface charged density to the surface concentration of the porphyrin molecules at the membrane. The obtained dependences of *R* on the surface concentration of porphyrins at the BLM were almost the same for all of the three investigated compounds (Figure 4). 

Moreover, if we plot, in the same figure, the similar dependence obtained earlier for the negatively charged sulfonatoporphyrin, TPPS4, it will be also in close proximity to those for the positively charged porphyrins. It allows us to conclude that the main factor determining the photodynamic efficiency of both positively and negatively charged porphyrins is their adsorption on the BLM. Other parameters, such as extinction or quantum yield of SO generation for all porphyrins, reveal much less difference. This conclusion is proved by the data of SO generation quantum yields of porphyrins, which depend slightly on the nature of the peripheral groups in the macrocycle [36]. 

### 3.3. Effect of pH on the Adsorption and Photodynamic Efficiency 

**Figure 5:**
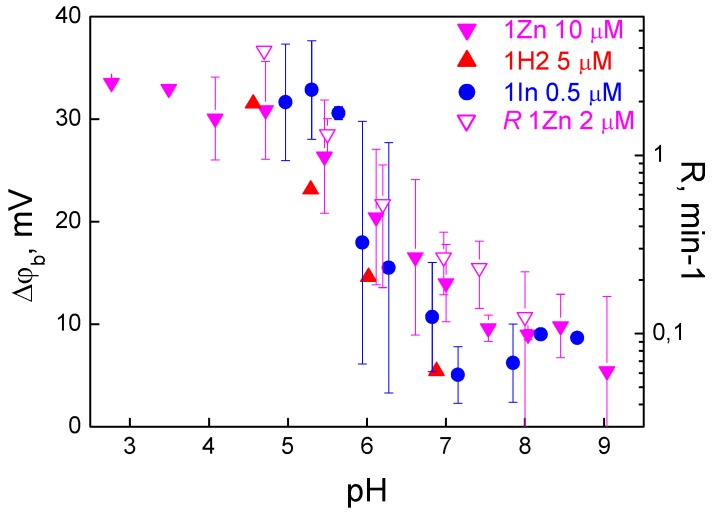
Dependence of the boundary potential due to adsorption of porphyrins (filled symbols, scale on the left) and the oxidation rate *R* of di-4-ANEPPS (blank symbols, scale on the right) on the pH. The measurements of Δφ_b_ are presented for the all three β-imidazolyl porphyrin derivatives, but the oxidation rate—only for 1Zn. Measurements were performed in a buffer solution containing 20 mM KCl, 2 mM HEPES, 2 mM Tris and 2 mM citrate. The pH value was changed by adding HCl or KOH to the solutions on both sides of the membrane.

In the experiments with all three porphyrins, we observed an increase in Δφ_b_ with a decrease in pH (Figure 5). 

This effect could be associated with a change in the surface charge of lipids; however, it has an opposite effect, because the membrane surface charge becomes more positive with a decrease in pH, which should suppress the adsorption of the cations. Another possible reason could be porphyrin penetration through the BLM. Penetration can depend on pH by the mechanism previously studied by us for remantadin [37] and proved later for hematoporphyrin [38]. Due to penetration, organic cations are present on both sides of the membrane, thus decreasing the Δφ_b_ measured by the IFC method. Such penetration becomes weaker at low pH, where the fraction of the neutral forms of the molecules is lower [34,35,37]. This mechanism can be tested by comparison of the Δφ_b_ measured by the IFC method with that determined by measuring the membrane conductance induced by nonactin. In the latter method, porphyrins were added symmetrically into the solutions on both sides of the membrane, and their possible penetration does not influence the value of the potential. The results presented in Figure 2 show that Δφ_b_ determined from the nonactin-induced change in conductance is the same as Δφ_b_ measured by the IFC method. This indicates that the penetration of these porphyrins can be neglected.

**Figure 6:**
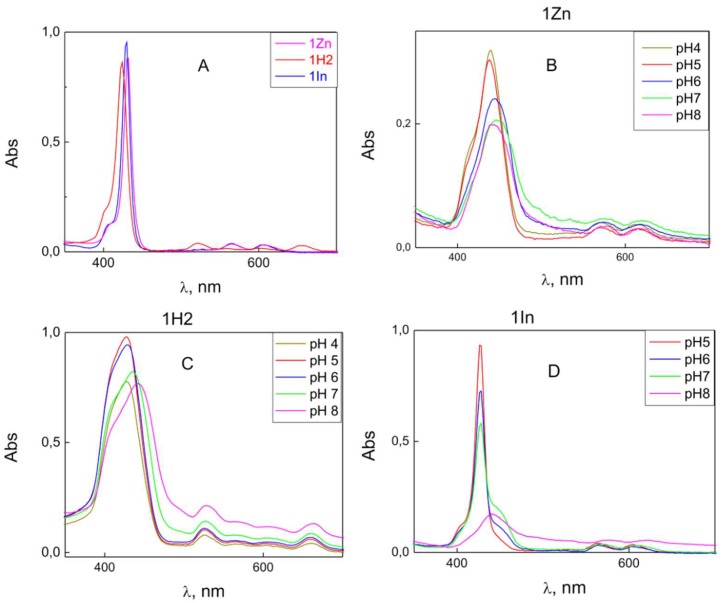
UV-VIS spectra of 5 μM solutions of β-imidazolium porphyrin derivatives in ethanol (**A**) and in water containing 20 mM KCl, 2 mM HEPES, 2 mM citrate and 2 mM Tris at various pH (1Zn—(**B**); 1H2—(**C**); and 1In—(**D**)).

Another possible explanation is the influence of pH on aggregation of porphyrin molecules in a water solution, which was observed earlier for other porphyrins. The aggregation of the porphyrins can be monitored by UV-Vis absorption spectroscopy (Figure 6).

The spectra of 1Zn and 1In dissolved in ethanol revealed a sharp Soret band with two Q-bands, while the spectrum of 1H2 showed four Q-bands, which was typical for free-base porphyrins. However, the spectra of water solutions were different and depended on pH and time. The spectrum similar to that in ethanol was observed only for 1In at low pH values. An increase in pH led to a decrease in the Soret band intensity; at pH 8, this band disappeared, while another band at a higher wavelength appeared. In the case of 1H2, the variation of the spectrum with pH was lower. For porphyrins 1In and 1Zn, the Soret band intensity decreased with time. The pH-induced changes in the spectrum were irreversible: coming back to lower pH values did not restore the spectrum observed initially at low pH. The decrease in the Soret band absorbance at high pH could be a result of aggregation of porphyrin molecules. The decrease in the absorption of 1Zn could also be a result of demetallation and transformation to 1H2. The pH also influenced the photodynamic efficiency of the porphyrins, as the oxidation rate of di-4-ANEPPS (Figure 5) increased with a decrease in pH. This indicates that pH influences the amount of the porphyrin molecules bound to the membrane.

The pH dependences of the membrane binding for positively charged porphyrins investigated here significantly differ from that of negatively charged porphyrin TPPS4 studied by us earlier [19]. The Δφ_b_ induced by positively charged porphyrins increases with a decrease in pH, in contrast to TPPS4, where Δφ_b_ decreases reaching zero at pH values lower than 3. The decrease of Δφ_b_ for TPPS4 is explained by its aggregation, which increases at low pH and thus prevents the adsorption of the TPPS4 molecules on the membrane. The same reason can explain the lack of adsorption of positively charged porphyrins at high pH values.

## 4. Conclusions

In this study, we attempted to evaluate the adsorption and oxidation rate of the positively charged β-imidazolium-substituted porphyrins on a bilayer lipid membrane for a better design of therapeutic molecules against skin cancer and drugs against antibiotic-resistant bacteria. It was found that the main factor determining the efficiency of these porphyrins is their adsorption on the membrane. The adsorption of the positively charged porphyrins is better than that of the negatively charged derivatives due to the surface charge of the lipid membranes, which is typically negative. The pH of the solution influences the adsorption of the positively charged porphyrins in contrast to the negatively charged ones: for positively charged porphyrins, the adsorption is enhanced at low pH, while for negatively charged porphyrins, the reverse dependence is observed. The most probable reason for this is the aggregation of porphyrin molecules in water. The better adsorption of the positively charged porphyrins can be important for their application as photosensitizers in photodynamic therapy against cancer cells whose membranes are negatively charged.

## Supplementary Materials

The following are available online at https://www.mdpi.com/2218-273X/9/12/853/s1, Figure S1: ^1^H NMR spectra of 1H2 and 1In in CDCl_3_. ^1^H NMR spectrum of 1Zn was recorded in CDCl_3_/MeOD mixture (1/1) in the presence of 2 equiv. DABCO. Figure S2. ^1^H NMR spectrum of 1In in CDCl_3_. Figure S3. ^13^C NMR spectrum of 1In in CDCl_3_. Figure S4. COSY spectrum of 1In in CDCl_3_. Figure S5. HR-MS spectrum of 1In.
