# Supplementary material for: Lipid Membrane Adsorption Determines Photodynamic Efficiency of β-Imidazolyl-Substituted Porphyrins
Source: Biomolecules. 2019 Dec 10;9(12):853. doi: 10.3390/biom9120853 (PMC6995582; doi:10.3390/biom9120853)
Supplement: Supplementary file 1 [file biomolecules-09-00853-s001.pdf]

Supplementary materials

Lipid membrane adsorption determines photodynamic efficiency of β-imidazolyl-substituted porphyrins

Irene Jiménez-Munguía [1,*], Arseniy K. Fedorov [2], Inna A. Abdulaeva [2], Kirill P. Birin [2], Yury A. Ermakov [2], Oleg V. Batishchev [2], Yulia G. Gorbunova [2,3,*] and Valerij S, Sokolov [2]

1 National University of Science and Technology ¨MISiS¨, 4 Leninskiy pr. 119049 Moscow, Russia

2 A. N. Frumkin Institute of Physical Chemistry and Electrochemistry, Russian Academy of Sciences, 31/4 Leninskiy pr. 119071 Moscow, Russia; fedorov@gmail.com (A.K.F.); Abdulaeva@gmail.com (I.A.A.); kirill.birin@gmail.com (K.P.B.), yury.a.ermakov@gmail.com (Y.A.E.), olegbati@gmail.com (O.V.B.), sokolov.valerij@gmail.com (V.S.S.)

3 Moscow Institute of Physics and Technology, 9 Institutskiy Lane, Dolgoprudniy, Moscow Region 141700, Russia

4 N. S. Kurnakov Institute of General and Inorganic Chemistry, Russian Academy of Sciences, 31 Leninskiy pr. 119119 Moscow, Russia

* Correspondence: sire.jm@hotmail.com (IJM), yulia@igic.ras.ru (YGG); Tel.: +7-495-955-4585

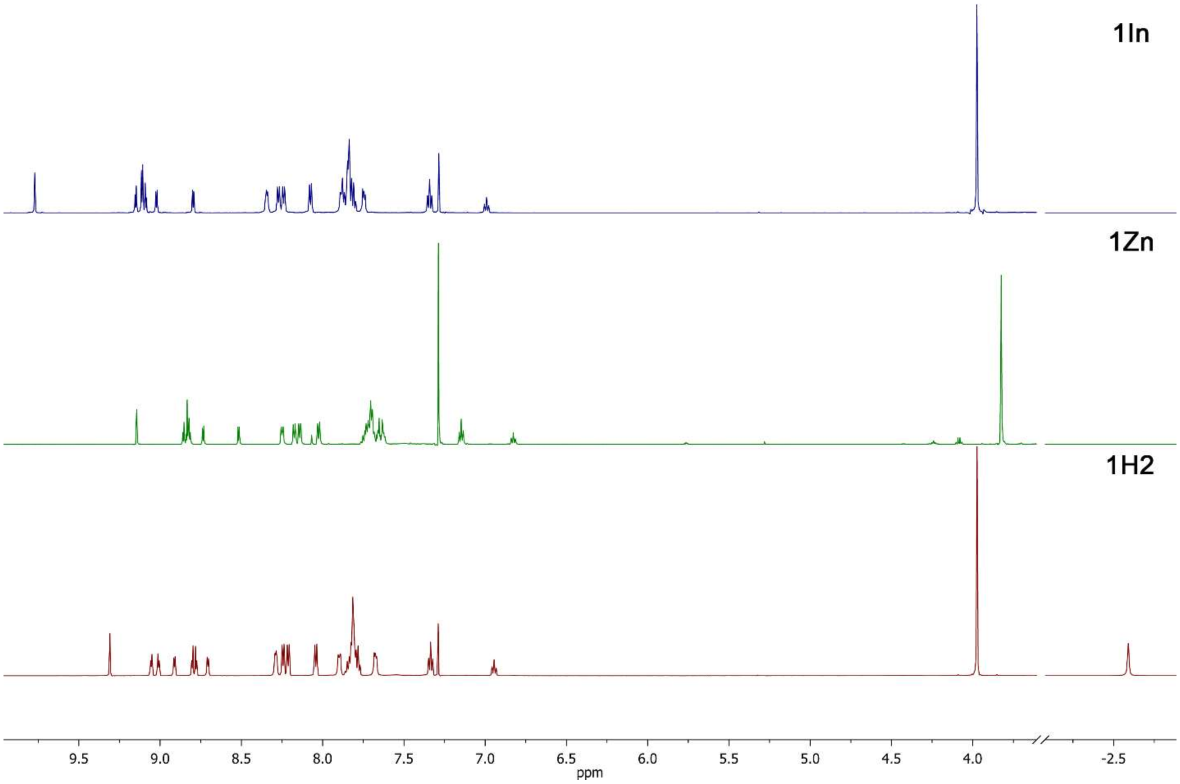

Figure 1. ^{1}H NMR spectra of 1H2 and 1In in $CDCl_3$. ^{1}H NMR spectrum of 1Zn was recorded in $CDCl_3$/MeOD mixture (1/1) in the presence of 2 equiv. DABCO.

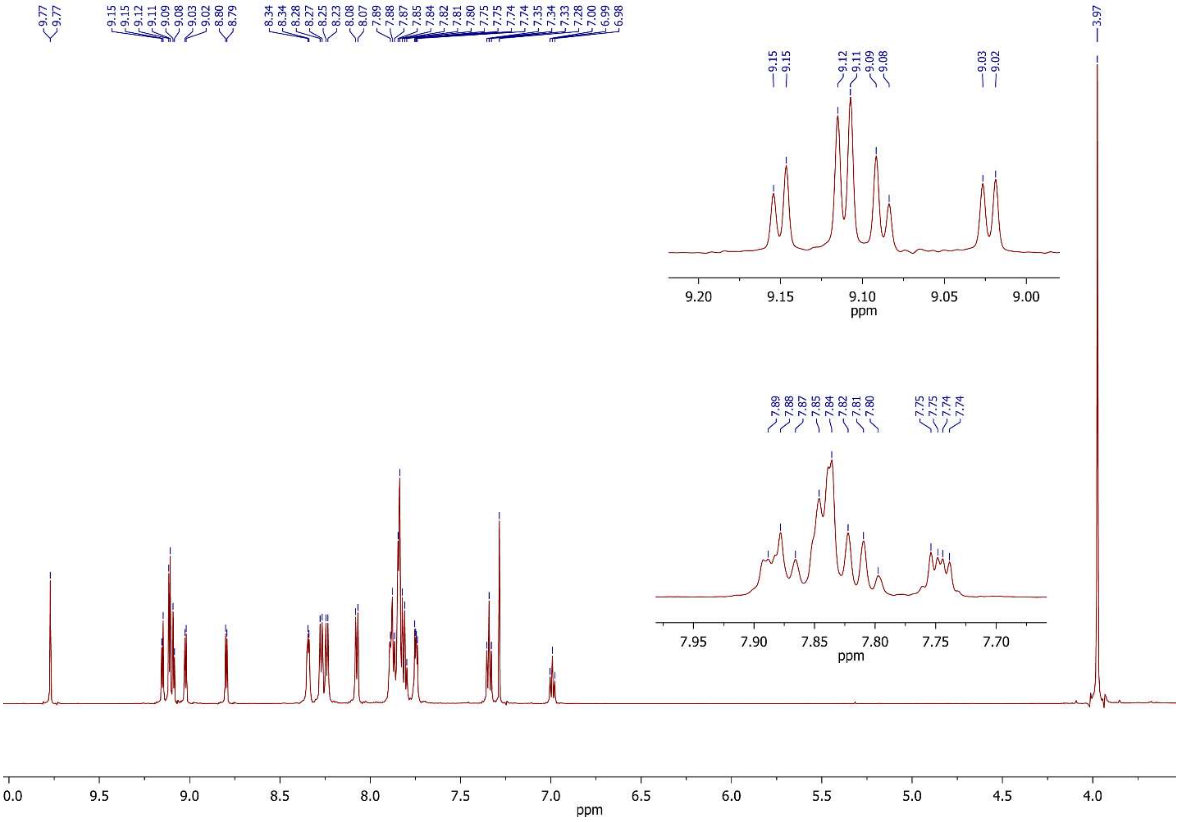

Figure 2. NMR spectrum of 1In in $CDCl_3$.

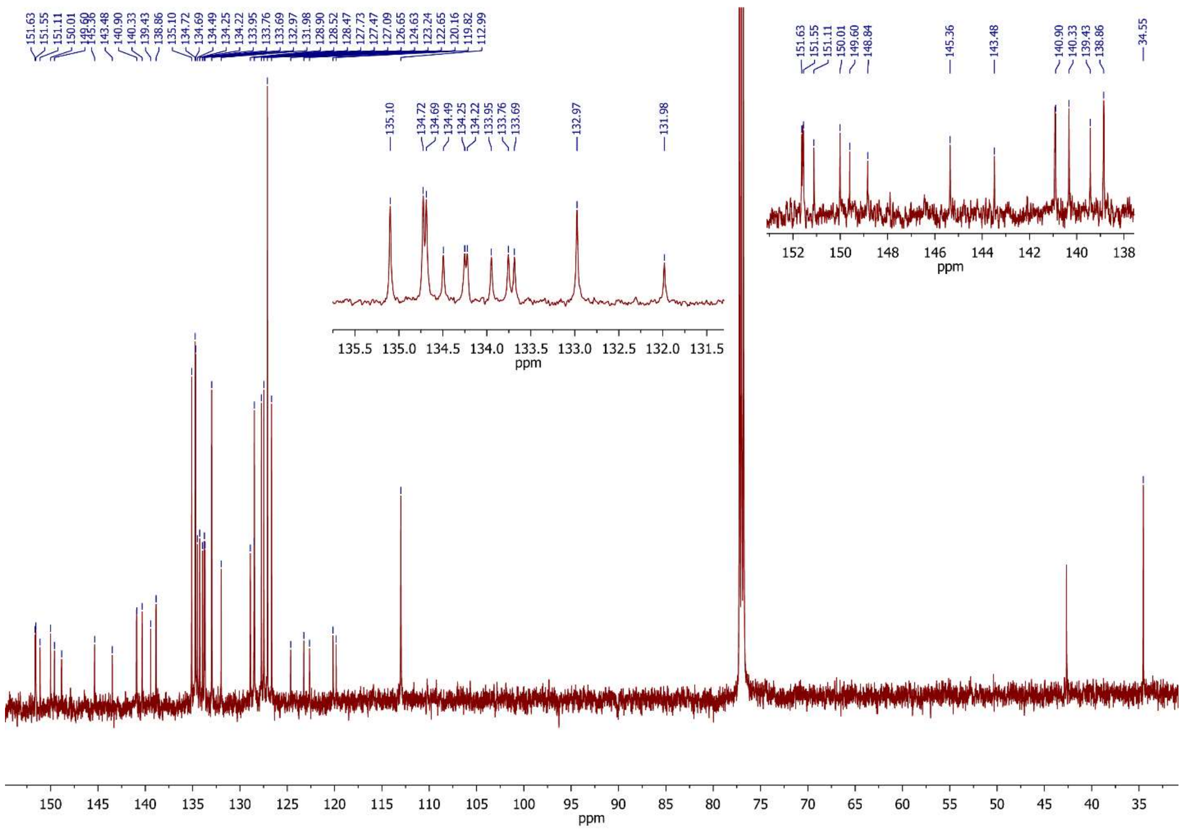

Figure 3. ^{13}C NMR spectrum of 1In in $CDCl_3$.

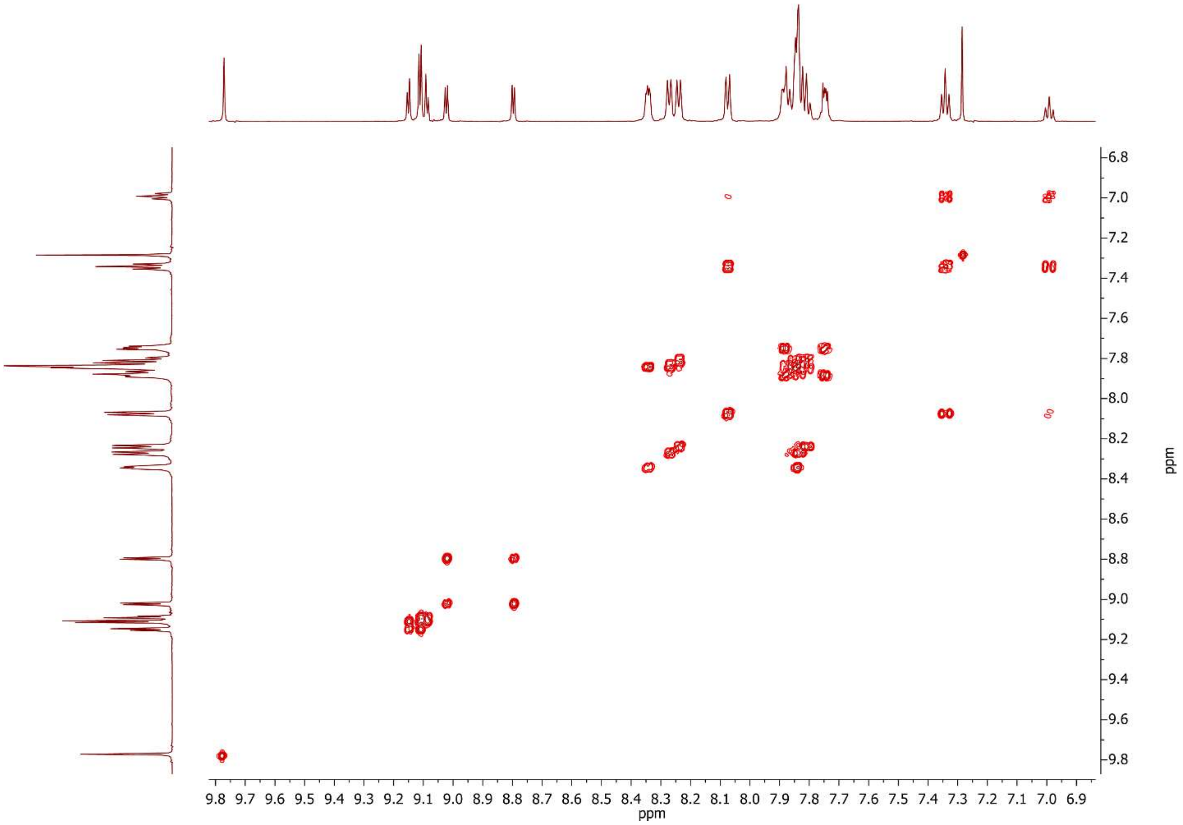

Figure 4. COSY spectrum of 1In in $CDCl_3$.

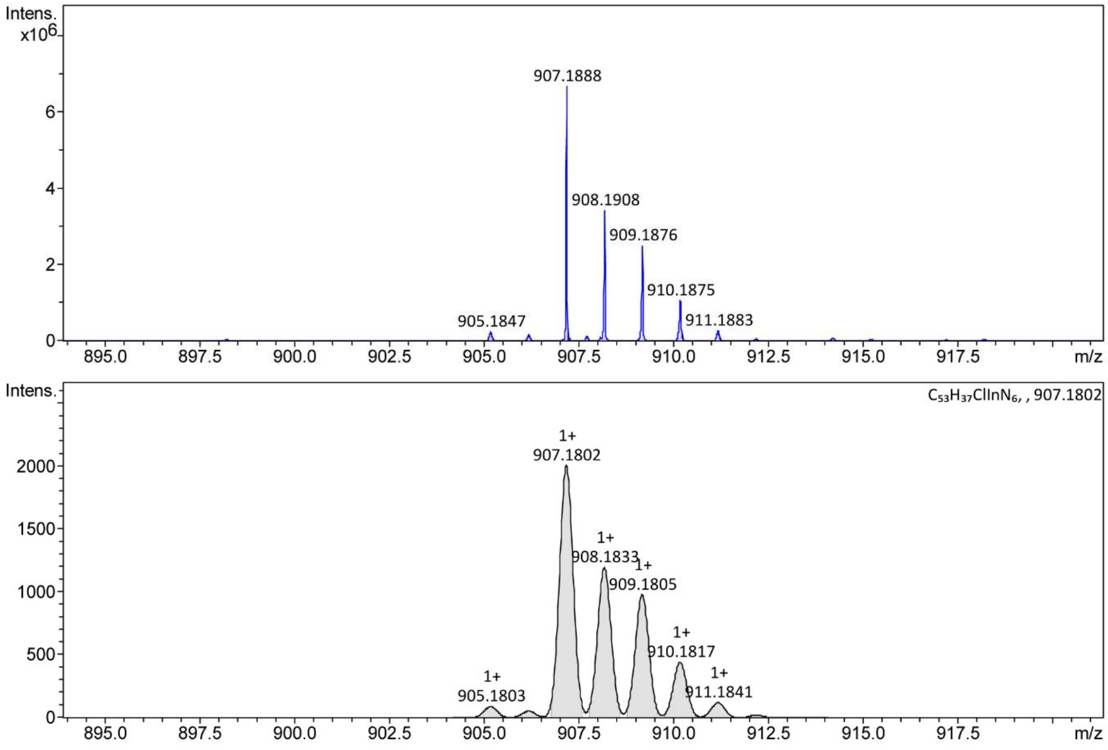

Figure 5. HR-MS spectrum of 1In.
